# Multi-Scale Clustering of Lyme Disease Risk at the Expanding Leading Edge of the Range of *Ixodes scapularis* in Canada

**DOI:** 10.3390/ijerph15040603

**Published:** 2018-03-27

**Authors:** Marion Ripoche, Leslie Robbin Lindsay, Antoinette Ludwig, Nicholas H. Ogden, Karine Thivierge, Patrick A. Leighton

**Affiliations:** 1 Department of Pathology and Microbiology, Faculty of Veterinary Medicine, University of Montréal, 3200 Rue Sicotte, Saint-Hyacinthe, QC J2S 2M2, Canada; patrick.a.leighton@umontreal.ca; 2 Epidemiology of Zoonoses and Public Health Research Unit (GREZOSP), Faculty of Veterinary Medicine, University of Montréal, 3200 Rue Sicotte, Saint-Hyacinthe, QC J2S 2M2, Canada; antoinette.ludwig@canada.ca (A.L.); nicholas.ogden@canada.ca (N.H.O.); karine.thivierge@inspq.qc.ca (K.T.); 3 Zoonoses and Special Pathogens Division, National Microbiology Laboratory, Public Health Agency of Canada, Winnipeg, MB R3T 2N2, Canada; robbin.lindsay@canada.ca; 4 Public Health Risk Sciences Division, National Microbiology Laboratory, Public Health Agency of Canada, 3200 Rue Sicotte, Saint-Hyacinthe, QC J2S 2M2, Canada; 5 Quebec Public Health Laboratory, Quebec Public Health Institute (INSPQ), 20045 Chemin Sainte-Marie, Sainte-Anne-de-Bellevue, QC H9X 3R5, Canada; 6 Institute of Parasitology, McGill University, 21111 Lakeshore Road, Sainte-Anne-de-Bellevue, QC H9X 3V9, Canada

**Keywords:** emerging disease, *Ixodidae*, tick distribution, nymph density, local scale, park, trail, heterogeneity

## Abstract

Since its detection in Canada in the early 1990s, *Ixodes scapularis*, the primary tick vector of Lyme disease in eastern North America, has continued to expand northward. Estimates of the tick’s broad-scale distribution are useful for tracking the extent of the Lyme disease risk zone; however, tick distribution may vary widely within this zone. Here, we investigated *I. scapularis* nymph distribution at three spatial scales across the Lyme disease emergence zone in southern Quebec, Canada. We collected ticks and compared the nymph densities among different woodlands and different plots and transects within the same woodland. Hot spot analysis highlighted significant nymph clustering at each spatial scale. In regression models, nymph abundance was associated with litter depth, humidity, and elevation, which contribute to a suitable habitat for ticks, but also with the distance from the trail and the type of trail, which could be linked to host distribution and human disturbance. Accounting for this heterogeneous nymph distribution at a fine spatial scale could help improve Lyme disease management strategies but also help people to understand the risk variation around them and to adopt appropriate behaviors, such as staying on the trail in infested parks to limit their exposure to the vector and associated pathogens.

## 1. Introduction

The emergence of vector-borne diseases in a new region creates a pressing need to understand the process of invasion for the vectors and factors underlying their rapidly changing distribution. Vector distribution is crucial information for vector-borne disease management, often providing the main source of data used to assess the epidemiological situation and the risk of transmission to humans, especially when the detection of human disease cases is difficult. Therefore, it helps to target prevention, surveillance, and control efforts [1]. 

The choice of the geographic scale is an important issue when measuring or mapping vector distribution [2]. Estimating the vector’s distribution at a broad spatial scale is an important step in obtaining an overall picture of the current and potential vector distribution. For instance, several studies have examined vector distribution as a function of current climate and under different scenarios of climate change [3,4]. This broad view is important for public health authorities to understand the epidemiological situation and to make decisions. However, such information is often not sufficiently refined to implement efficient local prevention strategies because fine-scale heterogeneity in habitat suitability or invasion history may result in local risk that differs substantially from risk measured at a broader scale [5]. 

At a broad spatial scale, the risk of exposure to a vector may be erroneously perceived as uniform over large geographic areas [1] and patterns on a finer scale may be hidden, especially where the area covers different ecological and climatic zones [5]. Microclimates and microhabitats can lead to important variation in vector distribution at a finer scale. In reality, high-risk areas commonly include pockets of low risk and, conversely, “islands” of high risk for vector exposure can occur within an “ocean” of low risk [1,6,7]. The issue of the spatial scale used to measure the risk and define control measures is particularly relevant in emerging areas where vector populations are not well established in all available suitable habitats. Consequently, there is a gap between the potential distribution based on suitable environmental conditions and the realized distribution, which is constantly evolving. This is the situation for Lyme disease in southern Canada where the invasion of the blacklegged tick, *Ixodes scapularis*, has been relatively recent and currently resulting in active range expansion [8,9]. 

*Ixodes scapularis* is the main vector of Lyme disease in the eastern part of North America. Lyme disease, which is caused by the bacteria *Borrelia burgdorferi sensu lato* complex [10], is the most frequent vector-borne disease in North America with around 30,000 cases reported in 2016 in the United States [11]. In the early 1990s, only one tick population was known in Canada, at Long Point in Ontario [12,13]. Since then, *I. scapularis* has expanded its geographic range northward into eastern and central Canadian provinces [14]. Human risk exposure to *B. burgdorferi* depends on the geographic distribution and abundance of *I. scapularis* [15,16]. *B. burgdorferi* distribution is closely linked to *I. scapularis* population expansion. The prevalence of *B. burgdorferi* within the tick population is very dynamic in the early phase of tick population establishment but stabilizes as tick populations reach equilibrium [1,6,17]. However, the knowledge of current tick distribution is the primary source of information used to assess potential human exposure to Lyme disease, and management of associated Lyme disease risk is mainly based on public and personal prevention. 

*Ixodes scapularis*’ spatial distribution has been studied at various geographic scales in North America [16]. Some studies reported *I. scapularis* tick distribution at a broad spatial scale, i.e., sampling sites in different counties or regions of one state [18,19,20,21,22], or sampling sites in the same county [23,24,25,26,27]. Some other studies were carried out at a local scale, i.e., sampling sites in the same city [16,27,28,29] or in the same forest or park [30,31,32,33,34]. The environmental drivers associated with the presence or abundance of *I. scapularis* also vary with the scale of the study. At broad spatial scales, tick distributions tend to reflect the presence of suitable habitat and climate conditions. Temperature and precipitation are the key factors delimiting the potential range of *I. scapularis* [35,36,37,38] and have been used to predict future range expansion under climate change [36]. However, at a local scale, tick population establishment is also influenced by the local interactions between environmental conditions and host distribution. Under appropriate climate conditions, *I. scapularis* preferentially lives in deciduous woodlands [39], such as mature oak or maple forests [40,41], although more recently maritime coniferous forests have also been found to be very suitable [14]. These environments should provide adequate temperature and humidity [15,40,42], depending in part on climate as well as the vegetation type and soil composition [2,39,41,43,44]. Moreover, the environment must also provide a suitable habitat for the host species that are key players in the tick’s life cycle, such as white-footed mice (*Peromyscus leucopus*) and white-tailed deer (*Odocoileus virginianus*) [29,45,46]. Previous studies at a local scale have tended to focus on environmental risk factors associated with tick occurrence, and relatively few studies have explored the determinants of local scale patterns of tick distribution for *I. scapularis* in North America [16] and for *Ixodes ricinus* in Europe [34,47].

In the province of Quebec, a key emergence zone for Lyme disease in southeastern Canada, the detection of an established tick population in a municipality relies on active surveillance, which consists of dragging a flannel sheet over 2000 m^2^ in a woodland within the municipality once during the summer to collect ticks in the environment [48,49,50]. Public health agencies use the results of active surveillance to attribute a risk level to each municipality [9,51]. The selection of sampling location for the 2000 m^2^ transect is by convenience and is usually close to a trail [52]. To date, studies on emerging tick distributions in Canada have focused on regional and provincial patterns; determinants of tick distribution at finer spatial scales have yet to been examined. These fine-scale processes are potentially important for the interpretation and use of active surveillance for building risk maps and improving the prevention strategies for Lyme disease. Is a sampled site representative of the other parts of the woodland? Are all parts of a woodland equally infested? It is also important for people who live, work, or visit areas invaded by ticks to be aware of the risk variation in these areas, making it possible for them to adopt appropriate behavior to limit their exposure to the vector and associated pathogens.

In this study, we explored for the first time multi-scale variation of Lyme disease risk at the leading edge of *I. scapularis*’ expanding range in Canada. We investigated tick distribution at three spatial scales within the Montérégie region, the primary Lyme disease emergence zone in southern Quebec [9]. We used spatial analysis and regression models to study the variation in nymph abundance and its associated ecological factors: (i) between different sampled woodland sites in the same region (site scale); (ii) between different sampling locations within the same woodland (plot scale); and (iii) at different distances from recreational trails (transect scale). 

## 2. Materials and Methods

### 2.1. Study Area

Montérégie is an agricultural region of 11,111 km^2^ [53] located in southwestern Quebec. It is bounded by the Saint Lawrence River to the north, the region of Estrie to the east, and the province of Ontario and the United States to the west and south. 

### 2.2. Sampling Protocol

**Figure 1:**
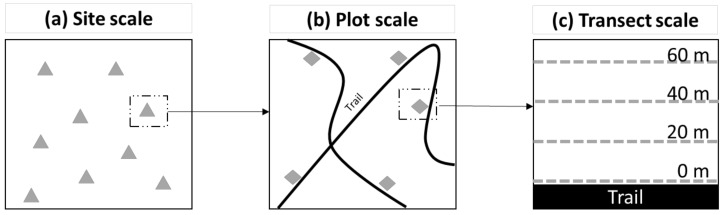
Sampling protocols at the site, plot, and transect scales. At the site scale (**a**), fifty sites (triangles) were sampled in 2014 by drag sampling an area of 2000 m^2^. At the plot scale (**b**), three public woodlands were intensively sampled in 2013 with several plots (diamonds) sampled along the trails (dark lines) during the summer. At the transect scale (**c**), four transects of 100 m (dotted lines) were sampled in each plot in 2013 at 0, 20, 40, and 60 m from the trail.

At site level, fifty woodlands were sampled across the Montérégie region in 2014 (Figure 1a). These sites had already been visited in 2007–2008 [52] for active surveillance of ticks in the environment. These sites were selected according to the presence of a suitable habitat based on the following criteria: zone of high or moderate risk for *I. scapularis* occurrence [4], deciduous (maple or mixed deciduous) woodland with minimal dimensions of 200 m × 200 m, ease of access, and owners’ authorizations [52]. Most sites were private woodlots. Each site was sampled once between May and September 2014, with a standardized effort of three person-hours of drag sampling (see below), equivalent to approximately 2000 m^2^ sampled per site [42].

For plot and transect scales, three public woodlands (Park 1, Park 2 and Park 3) were intensively sampled in 2013. These forest parks were chosen because they already had ticks in previous years (Park 1 and Park 2 since 2007, Park 3 since 2010), they were geographically close (about 10 km between parks), and they were popular public-access suburban nature parks. Each park was sampled weekly from May to August 2013. Ticks were collected in plots drawn from a random list of 100 potential plots located along the park’s trail network generated in ArcGIS version 10.3.1 (Environmental Systems Research Institute, Redlands, CA, USA) [54]. Plots consisted of four 100 m transects parallel to a park trail in a forested non-swampy area of the park (Figure 1b) placed at 0, 20, 40, and 60 m from the trail (Figure 1c). 

### 2.3. Tick Collection

Host-seeking ticks were collected by drag sampling, which consisted of dragging a 1-m^2^ flag of white flannel over the forest floor [55] in the morning or early afternoon. Drag sampling did not occur during rainfall or when the forest floor was wet. Every 25 m, the flag was inspected and ticks were removed and transferred to labeled vials containing 70% ethanol for transport to the Quebec Public Health Laboratory (LSPQ) for identification. Tick samples were identified to species and life stage using the taxonomic keys of Clifford et al. [56] (for larvae), Keirans et al. [57] (for nymphs), and Keirans and Clifford [58] (for adults). Nymph and adult *I. scapularis* were then shipped to the National Microbiology Laboratory (NML) where the *B. burgdorferi* infection status was determined using polymerase chain reaction (PCR) testing as previously described by Ogden et al. [59] and Bouchard et al. [52]. Testing was performed on individual nymphal and adult blacklegged ticks. To date, strains of *B. burgdorferi* detected in blacklegged ticks in Quebec are similar to those detected in other parts of North America [42].

### 2.4. Environmental Factors

Environmental variables were measured during the field visits or extracted from public georeferenced databases (see Table 1). Each field observation was carried out by the same field worker (M.R.) to minimize measurement and recording bias. The sampling period was divided into seasons as follows: “spring” for May and June; “summer” for July and August; and “autumn” for September, to account for the seasonality of tick stages. Environmental factors were collected at the site, the plot, and the transect scale (Table 1).

#### 2.4.1. Site Scale

Sites were characterized by the following ecological factors, estimated visually: percentage of canopy cover; percentage of forest floor area covered by ground vegetation, shrubs, and trees; presence of wetlands; and presence of woody debris on the forest floor. Litter depth was assessed by the average of measures taken at three points in the site. The average of the annual cumulative degree days > 0 °C and the total annual precipitation were calculated for each site [35] using data from Quebec meteorological stations from 2012 to 2014 [60]. The monthly mean temperature above 0 °C was multiplied by the number of days in a month and summed by years to obtain an estimate of the mean annual cumulative degree days >0 °C for each meteorological station. Monthly precipitations were summed by year and the mean total annual precipitation was calculated for each meteorological station. We then interpolated a raster surface from points using the inverse distance weighting (IDW) method with the Spatial Analyst Tool in ArcGIS (resolution = 5 km, power = 2, number of surrounding points = 12, using a variable search radius). We extracted the value of annual degree days > 0 °C and total annual precipitation from the interpolated surfaces for each site location [35].

#### 2.4.2. Plot Scale

Local temperature and relative humidity were monitored every 15 min during tick collection with an environmental data logger (HOBO H8 Pro series loggers, Onset Computer Corporation, Pocasset, MA, USA) set up in the middle of the plot, 30 cm above the soil. We calculated the arithmetic mean of temperature and percentage of relative humidity for each period of collection. The type of trail (soil, wood chips, or gravel/asphalt) and its width (mean of three measures at different points in the trail) were assessed for each plot. 

#### 2.4.3. Transect Scale

We visually characterized the vegetation height for each transect as low (e.g., grass), medium (e.g., ferns), high (e.g., shrub), or very high (e.g., mature trees). Litter type (soil, leaves, or conifer needles) and depth (mean of three measures in the middle of each transect) were assessed for each transect. 

### 2.5. Statistical Analysis

The statistical analysis focused on the density of nymphs because nymphs are the most important tick stage for the transmission of *B. burgdorferi* to humans [61,62] and are also the principal stage collected during drag sampling. The data from 2013 and 2014 were analyzed separately. Because of the low detection of *B. burgdorferi* in 2013 and 2014, we were not able to do this analysis for infected nymphs.

#### 2.5.1. Spatial Analysis

Spatial analyses were performed at the site and plot scale using ArcGIS version 10.3.1 [54]. Global Moran’s Index (Moran’s I) was used to assess the overall spatial clustering of *I. scapularis* distribution. Moran’s I was calculated for different distances with the *Incremental Spatial autocorrelation* in ArcGIS and presented in a spatial correlogram plotting values against the corresponding distance lag to determine the distance with the maximal effect (not presented here). A significant Moran’s I (*p* < 0.05) indicates the presence of significant spatial clustering of ticks in the environment, but the specific location of the clusters is not identified by this global index. 

We then used hot spot analysis to determine the locations of spatial clusters of high and low tick density. The Gi* statistic [63,64], a local indicator for spatial autocorrelations [65], gives an estimate for each point of its similarity to its neighbors compared to the whole sample [66]. Based on the z-score (z > |2|) and *p*-value (*p* < 0.05), a significant hot spot (or a cold spot) is a feature with a high (low) value and surrounded by other features with high (low) values as well. We used the *Hot Spot Analysis with Rendering* in ArcGIS with the inverse distance matrix for specifying the relationship between two sites. The threshold distance (d) to define neighbor sites was the optimal distance previously determined by Moran’s I correlogram [19] or the nearest neighbor distance for each point as determined by a proximity table calculated with the *Generate Near Table* in ArcGIS. 

#### 2.5.2. Regression Models

Count regression models were built using R software version 3.2.4 (R Development Core Team, Vienna, Austria) [67] to explore the relationship between nymph abundance and environmental factors at the site, plot, and transect scales. In each case, the response variable was the number of nymphs per sampling unit (site, plot, transect). We first selected explanatory variables with *p* < 0.20 in univariate analysis and then built the final model by a backward-stepwise selection procedure, conserving variables with *p* < 0.05 in the final model. To assess the fit of the model and the percentage of the variation in deviance explained by the model, we calculated McFadden’s pseudo R-squared with the following formula: 1—residual deviance/null deviance [68]. The season was constrained as a fixed term because nymph density and activity change over the summer, and there are more nymphs in spring than in summer and autumn [2]. Continuous factors were centered and scaled prior to analysis and correlations between factors were controlled in the final model with the variance inflated factor (VIF) to avoid collinearity [68]. Spatial autocorrelation of residuals was assessed using the global Moran’s I statistic. We tested both Poisson and negative binomial regression and selected the most appropriate approach based on the smallest Akaike information criterion (AIC) and the presence of overdispersion [69]. 

At the site scale, the number of nymphs per site in 2014 was modeled by negative binomial regression using the *glm.nb* function of the *MASS* package in R. As the tick collection at the site scale was not standardized on distance but rather on search effort (three person-hours), we added the log of sampling distance as an offset in the model. Because the global Moran’s I test indicated significant spatial autocorrelation in the residuals (*p* = 0.02), we added an autocovariate term to account for the spatial dependence of observations and improve the fit of the final model. This autocovariate term was calculated as an inverse distance-weighted function of the nymph abundance in neighboring sites [15,70,71] using the *autocov_dis* function of the *spdep* package in R. 

At the transect and plot scales, the number of nymphs per transect in 2013 was modeled as a multilevel model using a mixed-effect Poisson regression using the *glmer* function of the *lme4* package in R. Park and plot identity were added as random effects to reflect the hierarchical structure of the data: 251 transects within 63 plots within 3 forest parks. We used a random intercept model as we assumed that slopes were fixed but intercepts could change across groups. The global Moran’s I test showed no significant spatial autocorrelation in the residuals (*p* = 0.79) and we therefore did not include an autocovariate term in this model.

## 3. Results

### 3.1. Tick Collection

**Figure 2:**
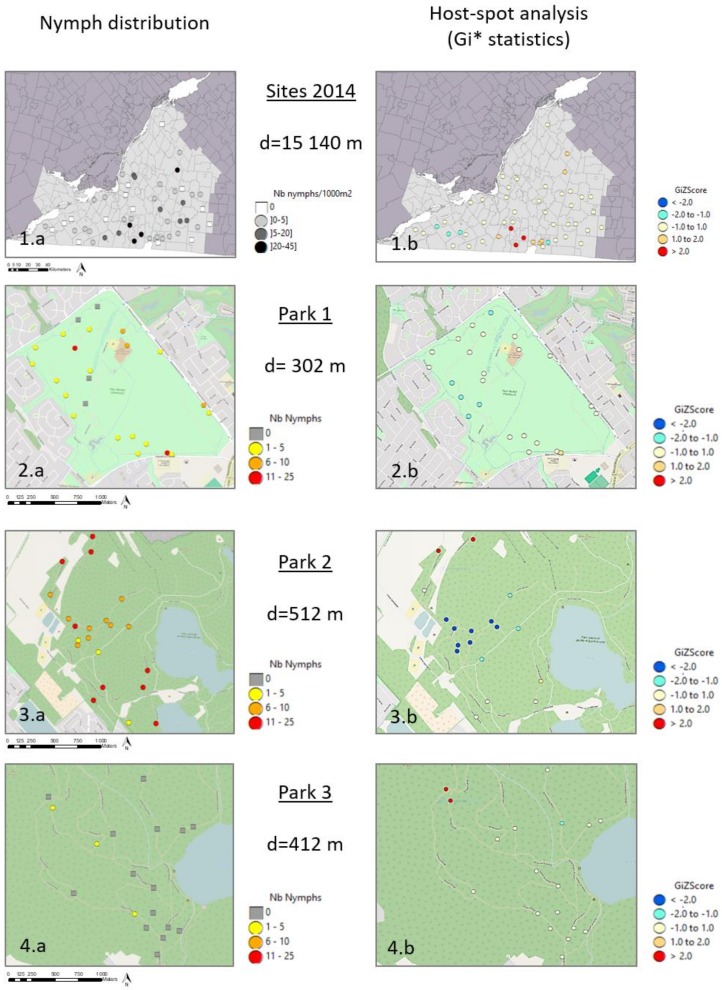
Density of host-seeking *I. scapularis* nymphs (**1a**–**4a**) and hot spot analysis (**1b**–**4b**) to detect local clusters of nymph density at site and plot scales. Moran’s I correlogram was used to determine the distance (d) used in the Gi* statistics. Significant hot spots (z-score > 2) are shown in red and significant cold spots (z-score < 2) in blue.

In 2014, *I. scapularis* was found in 86% of sampled sites with 1494 larvae, 342 nymphs, and 49 adults collected across all sites. Of these, 13% of nymphs and adults were infected by *B. burgdorferi*: 33 nymphs (10%) and 17 adults (35%), collected in nine sites. We noted a heterogeneous distribution of tick density and high variability among sites, ranging from 0 to 44 nymphs/1000 m^2^ (Table 2 and Figure 2(1a)). *B. burgdorferi*-infected ticks were found in the southern portion of the study area in sites with a high nymph density.

In 2013, *I. scapularis* was found in 71% of sampled plots and 53% of sampled transects: 210 larvae, 314 nymphs, and 7 adults. Only eight nymphs (2% of all collected nymphs and adults) were infected with *B. burgdorferi*, all collected in the same park (Park 2). Infected ticks were collected in six plots distributed throughout the park and at varying distances from the trail. We sampled 63 plots (24 in Park 1, 21 in Park 2, and 18 in Park 3) with four transects per plot, except for one plot in Park 1 where we could not sample the transect at 60 m from the trail because it was too close to another trail. There was considerable variation in the number of nymphs collected per plot (nymphs per plot: range = 0–25; mean = 5; 1st Qu. = 0; 3rd Qu. = 8). Although the three parks are geographically close (around 10 km) and all had ticks detected in the park prior to the study, the average tick densities differed greatly among the parks with 3.39 nymphs per plot (range: 0–14) in Park 1, 11.00 in Park 2 (range: 2–25), and only 0.22 in Park 3 (range: 0–2) (Table 2 and Figure 2(2a–4a)).

### 3.2. Spatial Distribution of Nymphs at Site Scale

The distance between two sites ranged from 2 km to 150 km. The global Moran’s I values indicated significant clustering of nymph density for sites in the Montérégie region, with a significant peak at the smallest lag of 15.14 km (Moran’s Index = 0.29, *p* < 0.001) but no significant autocorrelation beyond 20 km. The local Gi* statistic highlighted significant hot spots in south-central Montérégie close to the Richelieu River, and nonsignificant cold spots in the southwest (Figure 2(1b)) for the smallest distance that allowed neighbors between sites and with the best Moran’s I (d = 15.14 km).

At the plot scale, the distance between two plots ranged from 40 m to 2000 m. There was a global clustering for plots in Park 2 with a significant global Moran’s Index for distances from 400 to 800 m, with a peak at 512 m (Moran’s I = 0.30, *p* = 0.004). The local Gi* statistics identified significant hot spots and cold spots in Park 2. The global Moran’s Index was not significant for plots in Park 1 and Park 3. The local Gi* statistics identified significant hot spots in Park 3 but only nonsignificant cold spots in Park 1 (Figure 2(2b–4b)).

### 3.3. Relationship between Environmental Factors and Nymph Density

At the site scale, the final model explained 38% of the variation in deviance and included season, the square of elevation, litter depth, and a significant spatial autocovariate term (Table 3). Nymph density was higher in spring than in summer or autumn, increased by a factor of 1.58 [CI 95%: 1.01–2.55] for each centimeter of litter depth and decreased by 62% [CI 95%: 22%–83%] with the square of site elevation. The negative binomial distribution was better than the Poisson distribution due to overdispersion. The autocovariate term effectively removed spatial autocorrelation of the residuals (Moran’s I = −0.01, *p* = 0.79 for minimal distance of 15.14 km). It improved model fit (AIC = 281 with autocovariate term vs. 282 without it) but this difference did not change the ability of the model to fit the variation in the response variable [72]. The internal validity of the model was acceptable and removing the three outliers identified by Cook’s distance did not change the results of the model. The difference between observed and predicted nymph abundance (mean absolute error) was 11.26.

At the plot and transect scales, the final model was a mixed-effect Poisson regression including season, distance from the trail, type of trail, and the square of the relative humidity (Table 4). The model explained 19% of the variation in deviance. The park level explained 41% of the variation of the deviance and the plot level explained 10%. No autocorrelation was detected in the residuals of regression models for all distances tested (Moran’s I = −0.01, *p* = 0.79 for minimal distance). There were more nymphs in spring than in summer and the nymph densities were 1.85 [CI 95%: 1.17–2.36], 1.53 [CI 95%: 1.11–2.23], and 1.66 [CI 95%: 1.30–2.64] times higher at 60 m, 40 m, and 20 m than directly along the trail. However, there was no significant difference in nymph density between transects at 20, 40, and 60 m. There was 42% [CI 95%: 4%–64%] fewer nymphs in plots near gravel than near soil trails. The nymph density decreased by 26% [CI 95%: 7%–37%] with the increasing square relative humidity of the plot. The mean absolute error of the model was 0.95.

## 4. Discussion

In this study, we carried out the first multi-scale investigation of *I. scapularis* distribution and abundance in the environment in a recently invaded area in southern Canada. Our results provide evidence that tick distribution is not uniform and tends to be clustered at the site scale, the spatial scale used by active surveillance, but also at finer scales within woodlands sampled during active surveillance. This study highlighted natural environmental factors associated with nymph abundance, such as elevation, litter depth, and relative humidity, but also human-related environmental factors, such as distance from the trail and trail type, which are less commonly investigated. This study provides a portrait of the local distribution of ticks at the beginning of the invasion process and the factors highlighted could help to formulate recommendations for public health authorities for the interpretation and use of active surveillance results. This also could be useful for park managers and citizens to understand Lyme disease risk variation and improve local prevention strategies, such as identifying local risk areas or strengthening recommendations to stay on the trail in infested areas.

### 4.1. Regional Scale

We found that tick density was highly variable among woodlands within the newly invaded Montérégie region with evidence of clustering of high-density sites. Host-seeking nymph distribution at the site scale was heterogeneous and aggregated with spatial autocorrelation up to a distance of 20 km. There were some hot spots—clusters of sites with a high nymph density—in south-central Montérégie along the Richelieu River. This area was previously identified as a high-risk area in 2007 [38,73] with a significant cluster of established tick populations and emerging *B. burgdorferi* prevalence [42,74]. Similar studies conducted in the United States also found spatial autocorrelations of tick density and identified high-density clusters at a county scale [7,18,19,27,44,75,76,77]. In an endemic area, high-density clusters and spatial autocorrelation at small distances was suggested as signaling the presence of foci of early establishment and subsequent expansion from these focal areas [20]. It seems that this pattern is already visible in an emerging area such as southern Quebec. Hot spot analysis and spatial autocorrelation metrics allowed us to highlight the heterogeneity of tick distribution at the site scale and within a site, but clustering may also exist at an intermediate scale and could have been detected had we sampled sites that were closer together. Beyond spatial clustering, variation in nymph activity during the day and throughout the summer may have contributed to the heterogeneity in the observed tick density [2]. However, sites and plots were randomly distributed in time and space and with tick collection mostly done in the morning, and season was included as a fixed effect to account for the variation of nymph density during the summer. 

At the site scale, the heterogeneity of tick distribution is consistent with the process of invasion of *I. scapularis* in Quebec. The introduction of ticks to a new area may depend on both long-distance dispersal by migratory birds [4] and short-distance dispersal (<5 km) by resident hosts, especially deer transporting large numbers of adult ticks [78]. Tick distribution depends on the suitability of the environment, such as climate and habitat conditions, but also the presence and abundance of hosts. The variability of these factors results in heterogeneity in tick distribution and consequently in the spatial pattern of Lyme disease risk [2,44,77,79]. It was not the focus of our study to measure the impact of host movements and abundance, but some environmental factors were highlighted. The count regression model showed that nymph abundance increased with increasing litter depth. Litter depth is regularly cited as an important environmental factor for tick survival [73]. Ticks are more abundant where a thick leaf litter layer covers the ground, protecting ticks from desiccation [80,81,82] and from weather variation and winter cold [41,43]. Many previous studies have reported a negative effect of elevation on tick abundance [15,35,81,83,84,85,86,87]. We found that nymph abundance per site decreased with the square of elevation, which suggests a nonlinear relationship between nymph density and elevation. Diuk–Wasser et al. [15] also noted a nonlinear and negative association between altitude and the number of nymphs and found no nymphs above 510 m altitude in sampled sites in Eastern United States. Elevation may partly explain differences in tick density among the parks as the average elevation of plots in the low-density park (Park 3) was higher than in the others (average elevation: 214 m in Park 3 vs. 96 m in Park 2 and 35 m in Park 1). Litter depth and elevation have also been previously reported as predictors of the distribution of *I. ricinus*, a vector of Lyme disease and tick-borne encephalitis in Europe [88,89].

### 4.2. Local Scale

At a local scale within a single site, we also found significant variations in the number of nymphs collected per plot (range: 0–25 nymphs per plot) with a clustered distribution within the different sampled parks. This result observed in an emerging area is consistent with other studies of fine-scale nymphal *I. scapularis* distribution in endemic areas in northeastern United States [2,16,32] and for *I. pacificus* in California [33,90,91]. The similar patterns in endemic and emerging areas may be explained by the fact that this emerging area is in fact a combination of infested and not yet infested woodlands. Within infested parks, the clustered distribution suggests that some parts of the woodland are more suitable for *I. scapularis* nymphs in terms of environmental conditions and host activity. Pardanani and Mather [16] concluded that at this scale, nymph distribution is driven by microhabitat variables, microclimatic factors, and host dynamics. Talleklint–Eisen and Lane [91] suggested that nymphal clustering is likely in microhabitats favored by the hosts of the larval stage and where environmental conditions are suitable for tick development and survival. On the other hand, Ostfeld et al. [32] proposed that clustering results from microsite preferences by hosts and a synchronous detachment of fed ticks from the host. Our regression models supported the role of microclimate and also suggested the importance of host distribution in tick clustering distribution.

Nymph count per plot increased with the square of relative humidity. Relative humidity is regularly quoted as an important factor in other models of tick density [15,47,92,93], and arthropod vector density more generally [94,95]. Ticks need humidity around 80%–85% to be active and avoid desiccation [15,40,42,96]. Relative humidity depends on the vegetation, soil, slope, altitude, and is not homogeneous within a forest [2,39,41,43,44,97]. In our plots, relative humidity varied from 42% to 87%. In dry plots, ticks were not present or not detected because they were probably under cover to protect themselves from desiccation [41,43]. At the opposite extreme, very humid plots likely regularly accumulate standing water during the summer and were too humid to provide an optimal habitat for ticks [98]. Ticks are generally found in moist soils but not in poorly drained soils where standing water occurs. A previous study in Quebec found that *I. scapularis* abundance was associated with well-drained soils that effectively limited the excess of humidity [73]. 

No effect of temperature or height of vegetation on the number of nymphs was observed between or within sites. Although the effects of these factors on tick abundance are well known [41,99], the design of our study limited these effects because the vegetation and temperature were too similar between plots for statistical analysis. Some studies have also reported an effect of vegetation height or density in relation to the difficulty of drag sampling at these sites [100,101], but we did not observed this type of effect. Other studies have suggested investigating the vegetal community at fine scales [32,47,102] and forest fragmentation at broad scales [28,31,103], factors that would be interesting to explore in future studies.

We found that the distance from the trail (trail edge vs. off-trail) had a significant effect on nymph abundance, with a lower tick density near the trail than in the adjacent forest. A similar effect was also observed with *I. ricinus* in one forest park in France (<50 m vs. >50 m of the trail, [34]). However, our study was the first to observe the effect of trail proximity for *I. scapularis*. This result could have interesting applications for public health. On the one hand, local conditions may be better for ticks in the undisturbed woods with a closed canopy, tall vegetation, and leaf litter protecting them from desiccation than near the trail with a more open canopy, little or no leaf litter, and low vegetation. Werden et al. [104] found a positive correlation between canopy cover and the number of *I. scapularis* nymphs in Ontario. On the other hand, the trail may be a limiting factor for the movement of hosts that prefer to avoid areas disturbed by humans [34,105]. Moreover, we showed that there were more nymphs in a plot near a soil trail than in a plot near a gravel trail. To our knowledge, this is the first time that this relationship has been documented. Human activity and development may disturb the environment and alter host distribution, affecting tick distribution [105]. There are probably more gravel trails in the parts of parks more frequented by humans or accessible by vehicles, for instance near the entrance of the park. In recreational sites in the Northeastern United States, Falco and Fish [106] found that for most parks there were fewer *I. scapularis* nymphs in areas that are heavily used by the public. The authors suggested that the alteration of the habitat may have discouraged mammalian hosts or decreased tick survival. The location of hot spots and cold spots in our study supports the hypothesis of human disturbance: in sampled parks in 2013, hot spots were identified in less frequented parts of the park, while cold spots were at the entrance of the park, the entry point of the different trails, or next to residential neighborhoods. 

### 4.3. Implications for the Management of Emerging Lyme Disease Risk

The patchy distribution of *I. scapularis* raises a number of issues regarding the design, interpretation, and presentation of results of active tick surveillance. Indeed, the site scale with ticks collected in one site per municipality corresponds to the scale and the protocol used by the active surveillance program of the Public Health Institute of Quebec [107]. Drag sampling is the reference method used to collect ticks in the environment. Ogden et al. [108] estimated a sensitivity of 50% and a specificity of 86% to identify early-established *I. scapularis* populations using active surveillance, and suggested that one site visit of drag sampling consisting of three person-hours between May and October may be sufficient to identify a Lyme disease risk location; that is to say, the established *I. scapularis* population defined as the detection of the three stages and at least one infected nymph in the same year [107]. Another study estimated that only 10% of ticks present in the environment are collected during a site visit using this method [109]. Drag sampling remains the best method to approximate human exposure when walking in the woods. The standardization of this method makes it possible to compare the results between sampled locations. However, one visit of three person-hours only allows a limited portion of a woodland to be sampled. Our results and other studies show that caution must be used when extrapolating tick occurrence and abundance to another scale [16]. For instance, particularly in the situation of emerging tick populations where tick density is low, it may be difficult to attribute an accurate risk level to a municipality based on sampling a small part of one woodland within this municipality, especially if no ticks were collected. Because of the variability in tick density among similar habitats within the same park, we suggest sampling different parts of a park and different parks in the same municipality to limit the impact of fine-scale heterogeneity in tick distribution and activity on the interpretation of surveillance results. 

The patchy distribution of ticks at regional and local scales also has implications for risk communication to the public. The goal of active surveillance is to provide a portrait of the epidemiological situation. Risk maps produced must be useful for public health authorities and easily updated. However, scale and resolution of a risk map must be adapted to the message and intended audience [110]. Public health authorities generally choose to develop risk maps based on administrative units such as regions or municipalities. However, our study confirmed that the level of risk may be highly variable within such units, raising the question of how best to represent limited surveillance data on risk maps without causing these to be misleading. Depending on the spatial resolution used, the characterization of *I. scapularis* distribution and resulting risk perception will be different [2,5,16]. Public health authorities should be aware of the risk variation within these administrative unit limits and this should be taken into account when publishing and interpreting risk maps. Furthermore, Lyme disease is emerging in Québec and the epidemiological situation will change in the future. It will be interesting to monitor and understand the evolution of *I. scapularis* and *B. burgdorferi* distribution at the different geographic scales and in the context of changing host distribution and environmental conditions. 

Local heterogeneity in Lyme disease risk may also impact park managers and citizens. Fine-scale tick distribution maps could help park personnel manage Lyme disease risk at the park scale. Our study provides additional evidence to support the recommendation for visitors to remain on the park trails to limit their exposure to ticks [111]. In addition, using soil trails and less-frequented areas may increase the risk of contact with questing ticks. Park managers maintain trails (remove leaves, and vegetation on soil trails), develop recommendations, and give information to staff and visitors. They may identify the “at risk” areas in their park by sampling different parts of the park or by environmental assessment as in our study (type of trail, vegetation, human frequentation). Understanding the local distribution of ticks and the potential variation in Lyme disease risk would allow citizens to better understand the diversity of conditions encountered in each woodlot, to better interpret the risk information provided at broader scales (e.g., municipality, county, or region) by public health authorities, and to better understand the potential time lag or mismatch between the level of risk attributed to a municipality and finer scale risk (e.g., the presence of ticks in individual yards or woodlots).

## 5. Conclusions

Our study has provided evidence that tick distribution is not uniform at multiple scales within a newly invaded region and that the scale of measurement has important consequences for the accurate representation and communication of tick-borne disease risk. Taking into account the heterogeneous distribution of nymphs could help public health authorities improve surveillance and prevention strategies for Lyme disease, but could also help the public to better understand the local risk to which they are exposed and to adopt appropriate behavior to limit their exposure to the vector and its associated pathogens. This study was a unique opportunity to document the multi-scale geographic pattern of tick distribution and Lyme disease risk at the leading edge of *I. scapularis*’ expanding range in North America. Many of the patterns and processes identified in this study are likely to be common to other areas of newly established tick populations. The results of this study may therefore provide insight for other regions and municipalities adapting to increasing vector-borne disease risk as *I. scapularis* continues to expand its range across southern Canada and in parts of the United States.

## Figures and Tables

**Table 1:** Description of environmental variables used in regression models by geographic scale.

Scale	Explanatory Variable	Value	Min	1st Qu.	Median	Mean	3rd Qu.	Max	Source
Site (*n* = 50)	Sampling distance	Continuous (m)	650	1612	1912	1794	2100	2600	Field
	Elevation	Continuous (m)	13.58	49.21	61.69	94.39	116.29	384.00	GPS
	Annual degree days > 0 °C	Continuous (°C)	2132	3268	3331	3265	3370	3489	[60]
	Total annual precipitation	Continuous (mm)	506	848	933	940	1064	1258	[60]
	Litter depth	Continuous (cm)	1.00	3.00	4.00	3.76	5.00	8.00	Field
	Percentage of canopy cover	Category	0%: 0; 25%: 1; 75%: 24; 100%: 13						Field
	Percentage covered by ground vegetation	Category	0%: 10; 25%: 15; 75%: 12; 100%: 9						Field
	Percentage covered by shrubs	Category	0%: 18; 25%: 18; 75%: 9; 100%: 4						Field
	Percentage covered by trees	Category	0%: 2; 25%: 18; 75%: 29; 100%: 1						Field
	Wetlands	Yes/No	Yes: 16; No: 34						Field
	Woody debris on forest floor	Yes/No	Yes: 20; No: 30						Field
	Season	Category	Spring: 11; Summer: 32; Autumn: 7						Field
Plot (*n* = 63)	Elevation	Continuous (m)	11.18	34.73	84.72	107.20	184.00	300.50	GPS
	Local temperature	Continuous (°C)	15.17	19.32	22.79	22.79	25.91	32.49	Data logger
	Local relative humidity	Continuous (%)	42.65	55.22	63.16	65.74	77.65	88.55	Data logger
	Width of trail	Continuous (m)	1.40	2.30	3.10	3.02	3.60	6.30	Field
	Type of trail	Category	Soil: 17; Wood chips: 2; Gravel/Asphalt: 44						Field
	Season	Category	Spring: 24; Summer: 39						Field
Transect (*n* = 251)	Litter depth	Continuous (cm)	0.00	1.00	2.00	2.81	4.00	12.00	Field
	Litter of leaves	Yes/No	Yes: 11; No: 240						Field
	Litter of conifer needles	Yes/No	Yes: 42; No: 209						Field
	No litter (bare soil)	Yes/No	Yes: 15; No: 236						Field
	Ground vegetation (e.g., grass)	Yes/No	Yes: 59; No: 192						Field
	Medium vegetation (e.g., ferns)	Yes/No	Yes: 69; No: 182						Field
	Tall vegetation (e.g., shrub)	Yes/No	Yes: 20; No: 231						Field
	Very tall vegetation (e.g., mature trees)	Yes/No	Yes: 246; No: 5						Field

**Table 2:** Tick collection at site, plot, and transect scales.

Unit	Year	Number of Units with Ticks/Total Number of Units	Sampling Area per Unit	Nymphs Collected /1000 m^2^				
				Mean	SD	Median	Min	Max
Site	2014	43/50 (86%)	650 to 2600 m^2^	4.83	11.72	1.50	0	43.81
Plot	2013	45/63 (71%)	400 m^2^	12.45	15.10	5.00	0	62.50
Transect	2013	133/251 (53%)	100 m^2^	12.50	18.90	0.00	0	110.0

**Table 3:** Regression model for nymph counts per site in 2014. We used a negative binomial regression model with the log-transformed sampling distance as an offset.

Model 1	Estimate	Std. Error	z Value	Pr (>|z|)
Intercept	−5.449	0.663	−8.209	<0.001
Season				
Summer vs. Spring	−1.054	0.488	−2.157	0.031
Autumn vs. Spring	−1.589	0.678	−2.343	0.019
Autumn vs. Summer	−0.535	0.615	−0.870	0.384
Elevation *	0.711	0.397	1.789	0.073
Elevation^2^ *	−0.966	0.332	−2.907	0.003
Litter depth *	0.460	0.196	2.341	0.019
Autocovariate term	0.001	0.0004	2.968	0.002

* Scaled variable.

**Table 4:** Regression model for nymph counts per transect in 2013. We used a mixed Poisson regression model using park and plot identity as random effects.

Model 2	Estimate	Std. Error	z Value	Pr (>|z|)
Intercept	0.806	0.554	1.455	0.145
Distance from trail *				
*20 m vs. 0 m*	0.519	0.190	2.730	0.006
*40 m vs. 0 m*	0.435	0.193	2.249	0.024
*60 m vs. 0 m*	0.624	0.187	3.331	<0.001
Type of trail				
*wood chips vs. soil*	−0.966	0.619	−1.562	0.118
*gravel/asphalt vs. soil*	−0.537	0.258	−2.078	0.037
*gravel/asphalt vs. wood*	0.429	0.573	0.749	0.454
Relative humidity **	0.212	0.110	1.925	0.054
Relative humidity^2^ **	−0.267	0.109	−2.441	0.014
Season				
*summer vs. spring*	−0.413	0.189	−2.182	0.029

* No significant differences between transects at 20 m, 40 m, and 60 m; ** scaled variable.
